# Nitric Oxide Enhances Photosynthetic Nitrogen and Sulfur-Use Efficiency and Activity of Ascorbate-Glutathione Cycle to Reduce High Temperature Stress-Induced Oxidative Stress in Rice (*Oryza sativa* L.) Plants

**DOI:** 10.3390/biom11020305

**Published:** 2021-02-18

**Authors:** Harsha Gautam, Zebus Sehar, Md Tabish Rehman, Afzal Hussain, Mohamed F. AlAjmi, Nafees A. Khan

**Affiliations:** 1 Plant Physiology and Biochemistry Laboratory, Department of Botany, Aligarh Muslim University, Aligarh 202002, India; harshagautam99@gmail.com (H.G.); seharzebus5779@gmail.com (Z.S.); 2 Department of Pharmacognosy, College of Pharmacy, King Saud University, Riyadh 11451, Saudi Arabia; mrehman@ksu.edu.sa (M.T.R.); afihussain@ksu.edu.sa (A.H.); malajmii@ksu.edu.sa (M.F.A.)

**Keywords:** antioxidants, *Oryza sativa*, reactive oxygen species, sodium nitroprusside, stomata

## Abstract

The effects of nitric oxide (NO) as 100 µM sodium nitroprusside (SNP, NO donor) on photosynthetic-nitrogen use efficiency (NUE), photosynthetic-sulfur use efficiency (SUE), photosynthesis, growth and agronomic traits of rice (*Oryza sativa* L.) cultivars, Taipie-309 (high photosynthetic-N and SUE) and Rasi (low photosynthetic-N and SUE) were investigated under high temperature stress (40 °C for 6 h). Plants exposed to high temperature stress caused significant reduction in photosynthetic activity, use efficiency of N and S, and increment in H_2_O_2_ and thiobarbituric acid reactive substance (TBARS) content. The drastic effects of high temperature stress were more pronounced in cultivar Rasi than Taipie-309. However, foliar spray of SNP decreased the high temperature induced H_2_O_2_ and TBARS content and increased accumulation of proline and activity of ascorbate–glutathione cycle that collectively improved tolerance to high temperature stress more effectively in Taipie-309. Exogenously applied SNP alleviated the high temperature induced decrease in photosynthesis through maintaining higher photosynthetic-NUE and photosynthetic-SUE, activity of ribulose 1,5 bisphosphate carboxylase/oxygenase (Rubisco), and synthesis of reduced glutathione (GSH). The use of 2-4-carboxyphenyl-4,4,5,5-tetramethylimidazoline-1-oxy-3-oxide (cPTIO, NO scavenger) substantiated the study that in the absence of NO oxidative stress increased, while NO increased photosynthetic-NUE and photosynthetic-SUE, net photosynthesis and plant dry mass. Taken together, the present investigation reveals that NO increased heat stress tolerance and minimized high temperature stress adversaries more effectively in cultivar Taipie-309 than Rasi by enhancing photosynthetic-NUE and SUE and strengthening the antioxidant defense system.

## 1. Introduction

Rice (*Oryza sativa* L.) is the staple food of an estimated 3.5 billion people worldwide and has the third-largest cereal production with 741.5 million tons recorded in 2014 [1]. India ranks second worldwide in rice production, contributing about 20% of the world rice production [2,3]. According to Shahbandeh [4] there has been a slight increase in global rice consumption over the last several years. In the year (2008/2009) global consumption of rice was about 437.8 million metric tons, while in (2018/2019) it was about 490.27 million metric tons, and it is expected to further increase due to increase in population and urbanization. On the other hand, the increasing global average temperature has become a key concern for rice productivity worldwide. According to the fifth assessment report of (IPCC, 2018) on climate change, the total global emissions need to fall by 45% from 2010 levels by the year 2030 and it should reach a net-zero by about 2050 [5]. The ongoing climate change will severely affect the rice production and hence impact food security. The estimated reduction in the rice yield in India is about 4.5–9.0% by the year 2039 due to climate change scenarios [6].

Nitric oxide (NO) is known as a key signaling molecule in plants that controls growth and development. Sources of NO production in plant cells include various pathways like nitrite-dependent pathway [7], *L*-arginine dependent pathway [8], nitric oxide synthase (NOS)-pathway [9], and other enzymatic [10] and non-enzymatic pathways [11]. Exogenously applied or endogenously synthesized NO modulates the expression of several genes involved in signal transduction, photosynthesis, production and detoxification of reactive oxygen species (ROS), primary metabolism, apoptosis, and stress responses [12,13]. NO has been well documented for its remarkable function that it acts as an antioxidant under a wide range of stress responses including high temperature stress [14]. It has also been shown that the oxides of nitrogen, NO and nitrogen dioxide play an important role in the atmosphere, especially in the production and destruction of tropospheric and stratospheric ozone. In the stratosphere, they act as catalyst in ozone destruction reducing ozone levels, while in the troposphere, photolysis of nitrogen dioxide is the only known route for ozone formation [15]. Moreover, high NO levels can have negative effect on the ecosystem. The excessive application of products increasing NO concentration in atmosphere results in formation of ground level ozone, which can damage vegetation and also associated with adverse health effects.

Notwithstanding that NO has different functions, it mitigates high temperature-induced oxidative stress due to its ability to detoxify the ROS and maintain cellular redox homeostasis. Application of sodium nitroprusside (SNP; NO source) has been found to stimulate activity of superoxide dismutase (SOD), ascorbate peroxidase (APX), catalase (CAT), and peroxidase (POD) under high temperature (45 °C) in reed plant callus and played an important role in the metabolism of H_2_O_2_ [16]. While high temperature stress disturbs membrane thermo-stability and increases lipid peroxidation and electrolyte leakage in plants due to heat-induced oxidative burst [17], NO interacts with superoxide and hydroperoxyl radicals to act as a barrier and terminate their chain formation and ensure the protection of the membrane [18]. Several studies have shown that NO counteracts abiotic stress strongly by regulating sulfur (S)-assimilation [19,20,21]. It has been reported in mustard that NO stimulated S-assimilation and GSH production of Cd-exposed plants [22]. Stomatal closure under abiotic stress initiated by abscisic acid (ABA) and effected through a complex symphony of intracellular signaling in which NO played an important role [23]. However, NO at high concentrations may not have the same effects. NO donor S-nitroso-N-acetylpenicillamine (SNAP) application increased the aperture of stoma and caused stomatal opening in *Vicia faba* leaves [24]. The response of plants may differ depending on their sensitivity to NO. In general, there are several reports that show involvement of NO in plant physiological processes under normal condition and stress acclimation. However, the effects of NO in high temperature stress acclimation through its influence on use-efficiency of nutrients and the corresponding changes in the antioxidant activity, photosynthesis and agronomic traits are not available in the literature.

The objectives of this study were (i) to determine the response of high and low nutrient-use efficient rice cultivars to high temperature stress, and (ii) to evaluate the effect of exogenous SNP application on photosynthetic nitrogen and sulfur-use efficiency and to observe how this helped in mitigating high temperature stress induced negative effects on photosynthesis, growth, and agronomic traits of rice cultivars. We examined the influence of NO (as SNP) on the mechanisms of nitrogen and sulfur assimilation, photosynthetic nitrogen and sulfur use efficiency, photosynthetic characteristics, activity of enzymes of ascorbate-glutathione cycle, oxidative stress, osmolyte (proline) content, and growth and agronomic traits in rice cultivars, Taipie-309 and Rasi differing in nutrient-use efficiency under high temperature.

## 2. Materials and Methods

### 2.1. Plant Material, Growth Conditions, and Treatments

Healthy seeds of the rice (*Oryza sativa* L.) cultivars BPT-5204, Taipie-309, Pusa-44, Panvel, Rasi, MTU-1010, CR-Dhan-310, Nidhi, CR-Dhan-311, and Nagina-22 were procured from the Division of Agronomy, Indian Agricultural Research Institute (IARI), New Delhi, India. Seeds were surface sterilized with HgCl_2_ (0.01%) for 2 min, followed by a thorough and repeated washings with double-distilled water. Surface-sterilized seeds were soaked in distilled water for 12 h before sowing. The soaked seeds were randomly placed on filter paper covered Petri-plates for germination in an incubator at 30 °C saturated with deionized water. After 96 h, germinated seedlings were transferred in 23-cm diameter earthen pots filled with sufficient quantity of acid-washed sand. The pots were kept in the environmental growth chamber (Khera-Instruments, New Delhi) with an average day/night temperatures of 28 °C/22 °C (±3 °C), light/dark regime of 14/10 h with photosynthetically active radiations (PAR) of 430 µmol m^−2^ s^−1^ and relative humidity of 65 ± 5%. The plants raised in sand culture were supplemented with Hoagland nutrient solutions. The modified Hoagland nutrient solution (300 mL) containing 3.0 mM KNO_3_, 0.5 µM CuSO_4_, 2.0 mM Ca(NO_3_)_2_, 1.0 mM NH_4_H_3_PO_4_, 25 µM H_3_BO_4_, 2.0 µM MnCl_2_, 50 µM KCl, 20 µM ZnSO_4_, 20 µM Na_2_Fe-EDTA, and 0.5 µM (NH_4_)_6_Mo_7_O_24_ was initially supplied as 1/4^th^ strength to the seedlings, later increased to half strength at the 10^th^ day and the solution was changed every 3 days.

For the first experimentation, screening of rice cultivars was done based on the analysis of nutrient-use efficiency of primary mobile (nitrogen) and secondary immobile nutrient (sulfur) and photosynthesis, growth, and agronomic traits in rice cultivars to select high and low nutrient-use efficiency cultivars. Taipie-309 and Rasi were emerged as high nutrient-use efficient and low nutrient-use efficient cultivars, respectively. For the second experimentation, rice cultivars Taipie-309 and Rasi were selected, one set of plants was maintained at 35 °C (control), while another set of plants was subjected to 40 °C (high temperature stress) for 6 h daily for 15 days and were then allowed to recover at 35 °C and grown for the experimental period. Further, to investigate the role of NO in mitigating the adverse effects of high temperature stress, modulation of N and S assimilation, and plant performance, 30 mL of 100 µM concentrations of SNP was applied on foliage of control and high temperature treated plants with a hand sprayer at 20 days after transferring seedlings. Further, to substantiate the effect of NO in the alleviation of heat stress, 30 mL of 100 µM cPTIO 2-(4-carboxyphenyl)-4,4,5,5-tetramethylimidazoline-1-oxyl-3-oxide (NO scavenger) was added along with SNP in the presence of heat stress. A surfactant teepol (0.5%) was added with the control and SNP treatments. The observations were made on H_2_O_2_ content, photosynthetic-NUE, photosynthetic-SUE, net photosynthesis and plant dry mass. The treatments were arranged in a randomly blocked design with four replicates (n = 4) for each treatment. Plants were sampled at 30 days after germination (DAG) for recording different parameters and agronomic parameters were taken at harvest time.

### 2.2. Determination of Photosynthetic-NUE and Photosynthetic-SUE

Photosynthetic-nitrogen use efficiency was calculated by the ratio of net photosynthesis to N content per unit leaf area. Photosynthetic-sulfur use efficiency was calculated by the ratio of net photosynthesis to S content per unit leaf area.

### 2.3. Measurement of Gas Exchange, Chlorophyll Content, and Rubisco Activity

Infrared Gas Analyzer (CID-340, Photosynthesis System, Bio-Science, USA) was used for the measurements of gas-exchange parameters, i.e., net photosynthesis (Pn), stomatal conductance (gs), and intercellular CO_2_ concentration (Ci). All gas-exchange measurements were performed in fully expanded uppermost leaves, at atmospheric CO_2_, 380 ± 5 µmol mol^−1^, relative humidity, ~70%, photosynthetically active radiation (PAR), 800 µmol m^−2^ s^−1^, and atmospheric temperature, 32 ± 1 °C, in between 10:00 to 11:30 am.

Chlorophyll content or greenness was measured in intact leaves with the help of SPAD chlorophyll meter (SPAD 502 DL PLUS, produced by Konica Minolta, Japan) in the early morning hours and represented in SPAD values.

Rubisco activity was determined following the method of Usuda [25] by monitoring NADH oxidation at 30 °C at 340 nm during the conversion of 3-phosphoglycerate to glycerol 3-phosphate after the addition of enzyme extract to the assay medium. The details of the method is provided in the Supplementary file S1.

### 2.4. PS II Activity

Maximal quantum efficiency of photosystem II (PSII) (F_v_/F_m_) was measured on the 3^rd^ leaf from the top using a chlorophyll fluorometer (JUNIOR-PAM, Heinz Walz, Germany). Minimal fluorescence (F_o_) and maximum fluorescence (F_m_) were obtained by dark adapting the leaf samples for at least 30 min prior to determination. Weak measuring pulses (0.1 µmol m^−2^ s^−1^) and saturating pulse (>6000 µmol m^−2^ s^−1^) were used to measure F_o_ and F_m_, respectively. The variable fluorescence (F_v_) was calculated as the difference between F_o_ and F_m_. The maximal quantum efficiency of PSII was calculated as a ratio of F_v_ to F_m_.

### 2.5. Estimation of Proline

Proline content was estimated in leaves using the method of Bates et al. [26]. The details of the method are provided in the Supplementary file S1.

### 2.6. Determination of TBARS and H_2_O_2_ Content

Lipid peroxidation was determined by measuring the amount of malondialdehyde content produced by thiobarbituric acid reaction as described by Dhindsa et al. [27]. Hydrogen peroxide (H_2_O_2_) content was determined in leaf by using the method of Okuda et al. [28].

### 2.7. Determination of Leaf Nitrogen Content

Leaf nitrogen content was determined in acid-peroxide digested material using the method of Lindner [29]. A 10 mL aliquot of the digested leaf material was taken in a 50 mL volumetric flask. To neutralize the excess of acid and to prevent turbidity, 2 mL of 2.5 N sodium hydroxide (NaOH) and 1 mL of 10% sodium silicate (Na_2_SiO_3_) solutions were added. Double distilled water (DDW) was added to make the volume up to the mark. In a 5 mL aliquot of this solution, 0.5 mL Nessler’s reagent was added in a 10 mL graduated test tube. The contents of the test tubes were allowed to stand for 5 min for maximum color development. Then, optical density (OD) value of the solution was read on a spectrophotometer at 525 nm. Leaf N content was calculated using graph plotted between different concentrations of ammonium sulphate and OD.

### 2.8. Determination of Leaf Sulfur Content

The leaf sulfur content was determined by using the method of Chesin and Yien [30]. Oven-dried leaf powder (100 mg) was transferred to 75 mL digestion tube. Acid mixture (4 mL) was prepared using concentrated HNO_3_ and 60% HClO_4_ in the ratio of 85:1 (*v*/*v*), 7.5 mg of selenium dioxide was added as a catalyst in digestion tube. The mixture was digested till the colorless solution obtained and volume was made up to 75 mL using DDW. The interference of silica was checked by filtering the contents of the tube. A 5 mL aliquot was transferred from the digestion tube to 25 mL volumetric flask for turbidity development. Turbidity development was initiated by adding 2.5 mL gum acacia (0.25%) solution, 1.0 g BaCl_2_ sieved through 40–60 mm mesh and the volume was made 25 mL with DDW, shaken thoroughly to dissolve BaCl_2_ completely. The turbidity was allowed to develop for 2 min. The values were recorded at 415 nm within 10 min after turbidity development. A blank was also run simultaneously after each set of determination, and calculation was done accordingly.

### 2.9. Determination of Content of Cysteine and GSH

Cysteine (Cys) content was determined by the method of Gaitonde [31]. Fresh leaf tissue (500 mg) was homogenized in 5% (*w*/*v*) ice-cold perchloric acid. The homogenate was centrifuged at 2800× *g* for 1 h at 5 °C and the supernatant was filtered using Whatman No.1 paper. The filtrate of 1 mL was treated with acid ninhydrin reagent. Finally, the absorption was read at 580 nm and Cys amount was calculated with reference to a calibration curve obtained for standard Cys.

The content of reduced glutathione (GSH) was determined according to the method described by Anderson [32]. Fresh leaf tissue (0.5 g) was ground in 2.0 mL of 5% sulphosalicylic acid under cold conditions. The ground tissue was centrifuged at 10,000× *g* for 10 min. 0.6 mL of phosphate buffer (100 mM, pH 7.0) and 40 mL of 5, 5-dithiobis-2-nitrobenzoic acid (DTNB) were added to 0.5 mL of supernatant. After 2 min the absorption was read at 412 nm, and calculation was done accordingly.

### 2.10. Assay of Antioxidant Enzymes

Fresh leaf tissue (200 mg) was homogenized with an extraction buffer containing 0.05% (*v*/*v*) Triton X-100 and 1% (*w*/*v*) polyvinyl pyrrolidone (PVPP) in potassium-phosphate buffer (100 mM, pH 7.0) using chilled mortar and pestle. The homogenate was centrifuged at 15,000× *g* for 20 min at 4 °C. The supernatant obtained after centrifugation was used to assay the activity of superoxide dismutase (SOD) by the method of Beyer and Fridovich [33] and Giannopolitis and Ries [34] and glutathione reductase (GR) by the method of Foyer and Halliwell [35], and for the assay of ascorbate peroxidase (APX), extraction buffer was supplemented with 2.0 mM ascorbate and was determined by adopting the method of Nakano and Asada [36]. The details of the assay of activity of antioxidant enzymes are given in the Supplementary file S1.

### 2.11. Activity of Nitrate Reductase

Leaf nitrate reductase (EC 1.7.99.4) activity was measured by using the method of Kuo et al. [37]. The details of the method are given in the Supplementary file S1.

### 2.12. Determination of NO Generation

NO generation was determined by adopting the method of Zhou et al. [38] by estimating nitrite content. Fresh leaf samples (500 mg) were homogenized in 3.0 mL of 50 mM ice-cold acetic acid buffer (pH 3.6) containing 4% zinc acetate using chilled mortar and pestle. The homogenate was centrifuged at 11,500× *g* for 15 min at 4 °C and the supernatant was collected. The pellet obtained was washed twice with 1.0 mL of the extraction buffer and then centrifuged again. The supernatant obtained from the two spins were combined and neutralized by adding 100 mg of charcoal. The filtrate was collected, after vortex and filtration. One mL each of the filtrate and Greiss reagent (1% sulphanilamide and 0.1% N-1-napthylethylenediaminedihydrochloride in 5% H_2_PO_4_ solution) was mixed in the ratio (1:1), and then for 30 min incubated at room temperature. The absorbance of the reaction mixture was read at 540 nm and NO content was estimated from a calibration curve plotted using sodium nitrite as standard.

### 2.13. Determination of Growth Characteristics and Agronomic Traits

Plants were harvested randomly to measure growth parameters and agronomic traits. To measure the plant dry mass (PDM), root and shoot samples were dried in an oven at 80 °C for 72 h till constant mass was obtained. The dried leaves were used for determining other parameters and the dry mass of root and shoot sample was taken to record PDM. Leaf area was measured with a leaf area meter (*LA211, Systronics,* New Delhi, India). The number of tillers per plant was counted manually at weekly intervals as well as at harvest. Daily observations were made for the appearance of panicles. The number of panicle per plant, panicles length, number of grains per panicle was determined manually at harvest time.

### 2.14. Scanning Electron Microscopy

Leaf samples were prepared for scanning electron microscopy (SEM) investigation according to Daud et al. [39] with slight modifications. The leaf samples used for observing the stomatal behavior were collected from the youngest fully expanded leaves. Subsequently, leaf samples were first fixed with 2.5% glutaraldehyde plus 2% paraformaldehyde in 0.1 M phosphate buffer (pH 7.0) in equal quantity for more than 4 h and then washed three times with phosphate buffer for 15 min at each step. The samples were then post fixed with 1% osmium tetraoxide in phosphate buffer (pH 7.0) for 1 h and washed three times with the same phosphate buffer for 15 min. After that, firstly, the specimens were dehydrated by a graded series of ethanol (50, 70, 80, 90, 95, and 100%) for about 15-20 min at each step, transferred to the mixture of alcohol and isoamyl acetate (*v*/*v* = 1) for about 30 min. Then, the samples were transferred to pure iso-amyl acetate for 1 h. In the end, the specimens were dehydrated in Carl Ziess EVO 40 (Germany) scanning electron microscope at extra high tension or high voltage at 15 kV and magnification of 1000× and 3000×. The leaf stomatal frequency was expressed as the number of stomata per unit leaf area, here stomatal frequency was determined by counting the number of stomata by circling 1 mm^2^ area in the microscope field of view. The stomatal aperture length and width were measured by micrometer scale.

### 2.15. Statistical Analysis

Data obtained were examined statistically using analysis of variance (ANOVA) with SPSS software version 17.0 for Windows and presented as a treatment mean ± SE (n = 4). The least significant difference (LSD) was calculated for the significant data at *p* < 0.05. Bars with the same letter are not significantly different by LSD test at *p* < 0.05. ANOVA Model for data presented in Tables and Figures is given in the Supplementary file S1.

## 3. Results

### 3.1. Screening of Cultivars

Table 1 shows that there was significant difference among rice cultivars in N and S assimilation, photosynthetic-NUE and photosynthetic-SUE, leaf area, and plant dry mass. The cultivar Taipie and Rasi exhibited highest and lowest values, respectively for photosynthetic-NUE and photosynthetic-SUE and plant dry mass. The cultivars also significantly differed in photosynthetic characteristics; net photosynthesis, intercellular CO_2_ concentrations, stomatal conductance, chlorophyll content (SPAD value) and maximal PSII photochemical efficiency (Table 2). Among the cultivars, Taipie-309 showed maximum values for photosynthetic characteristics, whereas Rasi exhibited minimum photosynthesis. Results of agronomic traits such as number of tillers and panicles per plant, length of panicles, and number of grains per panicle are presented in (Table 3).

The rice cultivars differed significantly in their abilities in producing tillers and panicles, variations in length of panicles, and number of grains per panicle. The Taipie-309 recorded the highest number of tillers and panicles, length of panicles, and number of grains per panicle, while Rasi recorded the lowest. Thus, among ten rice cultivars tested, Taipie-309 and Rasi emerged as the high and low photosynthetic-NUE and photosynthetic-SUE cultivars, reflecting high and low dry mass and yield traits, respectively. The order in which different cultivars showed photosynthetic-N and SUE, growth, and yield was: Taipie-309 > CR-DHAN-310 > BPT-5204 > Nagina-22 > MTU-1010 > Pusa-44 > Panvel > Nidhi > CR-DHAN-311 > Rasi.

### 3.2. Effect of NO on High Temperature Stress-Induced Oxidative Stress

**Figure 1:**
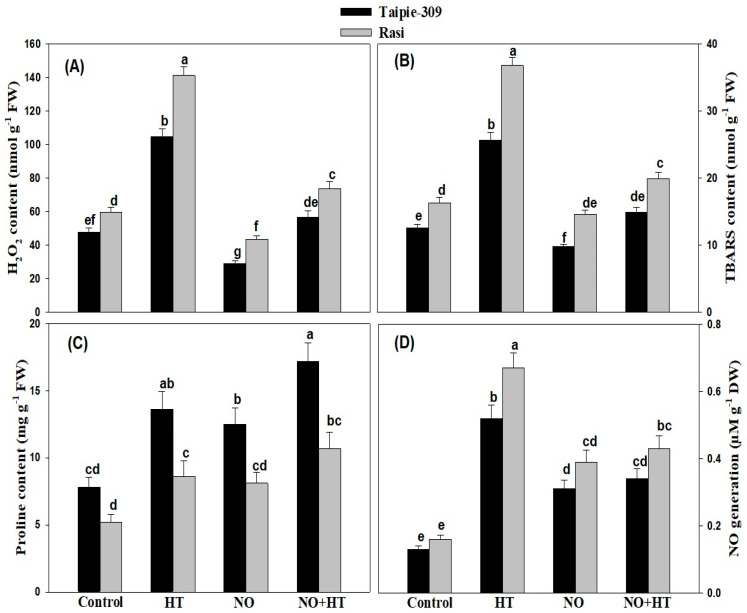
Leaf H_2_O_2_ content (**A**), TBARS content (**B**), Proline content (**C**), and NO generation (**D**) in Taipie-309 and Rasi cultivars of rice (*Oryza sativa* L.) at 30 days after germination (DAG), plants were grown with/without high temperature stress and treated with foliar 100 µM SNP (NO donor). Data are presented as treatments mean ± SE (n = 4). The values followed by same letters above bars represent that data did not differ significantly by LSD test at *p* < 0.05. FW, fresh weight; DW, dry weight.

To study the influence of NO in alleviation of high temperature stress-induced oxidative stress, we analyzed oxidative stress as content of TBARS and H_2_O_2_ following treatment by 100 µM SNP in the presence or absence of stress. As a response of high temperature, data presented in (Figure 1A,B) indicated that high temperature significantly increased H_2_O_2_ and TBARS content by 120.3% and 103.9% in Taipie-309 and 136.1% and 125.7% in Rasi, respectively, compared to the control plants. Our results indicated that application of 100 µM SNP significantly decreased H_2_O_2_ and TBARS content in Taipie-309 and Rasi by 38.6% and 22.2% and 27.2% and 10.4%, respectively, compared to the control plants. However, application of 100 µM SNP under high temperature condition restricted the H_2_O_2_ production and lipid peroxidation compared to the heat stressed plants, more conspicuously in Taipie-309 than Rasi.

### 3.3. Effect of NO Treatment on Proline Accumulation under High Temperature Stress

The effects of SNP application on proline accumulation in Taipie-309 and Rasi are presented in (Figure 1C). High temperature stress caused increase in proline content by 74.3% in Taipie-309 and 65.3% in Rasi compared to their respective control plants; nevertheless the maximum value was recorded in Taipie-309 cultivar. Additionally, under no stress condition 100 µM SNP increased proline accumulation in Taipie-309 by 60.2% and in Rasi by 55.7% compared with the control. Proline accumulation enhanced with the application of 100 µM SNP under high temperature condition, significantly increased proline content in Taipie-309 by 120.5% and in Rasi by 105.7% in comparison to the control plants.

### 3.4. Effect of NO on NO Generation under High Temperature Stress

High temperature treatment resulted in enhanced NO generation in comparison to the control in both cultivars, but Rasi exhibited greater accumulation of NO than Taipie-309. Application of SNP suppressed NO generation in both cultivars, thus stress-induced NO generation under high temperature responsible for high temperature induced negative effects on plant metabolism were suppressed. SNP treatment reduced NO generation by about 138.4% in Taipie-309 and 143.7% in Rasi compared to the control under no stress condition. Supplementation of SNP to high temperature treated plants reduced NO generation by about 168.7% and 161.5% in Rasi and Taipie-309, respectively compared to high temperature treated plants (Figure 1D).

### 3.5. NO Modulates Activity of Enzymatic Antioxidants under High Temperature Stress

**Figure 2:**
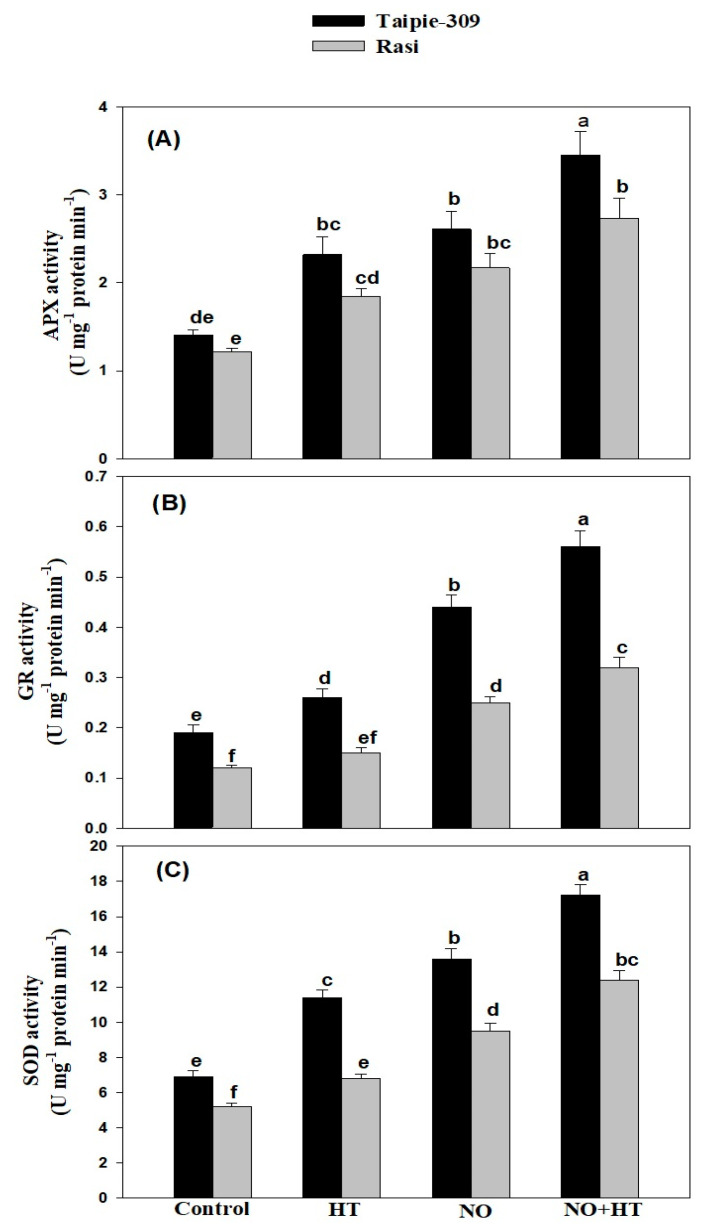
Ascorbate peroxidase (APX, **A**), glutathione reductase (GR, **B**), and superoxide dismutase (SOD, **C**) activities in Taipie-309 and Rasi cultivars of rice (*Oryza sativa* L.) at 30 days after germination (DAG), plants were grown with/without high temperature stress and treated with foliar 100 µM SNP (NO donor). Data are presented as treatments mean ± SE (n = 4). The values followed by same letters above bars represent that data did not differ significantly by LSD test at *p* < 0.05.

The enzymatic activity of three key antioxidants (SOD, APX, and GR) was evaluated. Antioxidant enzymes exhibited a marked increment in their activities under high temperature stress (Figure 2). In comparison to the control plants, a significant increase in APX (64.5% and 50.8%), GR (36.8% and 25.0%), and SOD (65.2% and 30.7%) activity was observed in the leaves of high temperature-stressed Taipie-309 and Rasi cultivars, respectively. The exogenous application of 100 µM SNP further stimulated the APX (85.1% and 77.8%), GR (131.5% and 108.3%), and SOD (97.1% and 82.6%) activity in Taipie-309 and Rasi, respectively as compared to the control plants. Similar enhancement was observed for APX (144.6% and 123.7%), GR (194.7% and 166.6%), and SOD (149.2% and 138.4%) activity in 100 µM SNP treated plants of Taipie-309 and Rasi, respectively under high temperature conditions compared to the control plants.

### 3.6. NO Alleviates the Adverse Impacts of High Temperature Stress on Growth and Photosynthetic Traits

**Figure 3:**
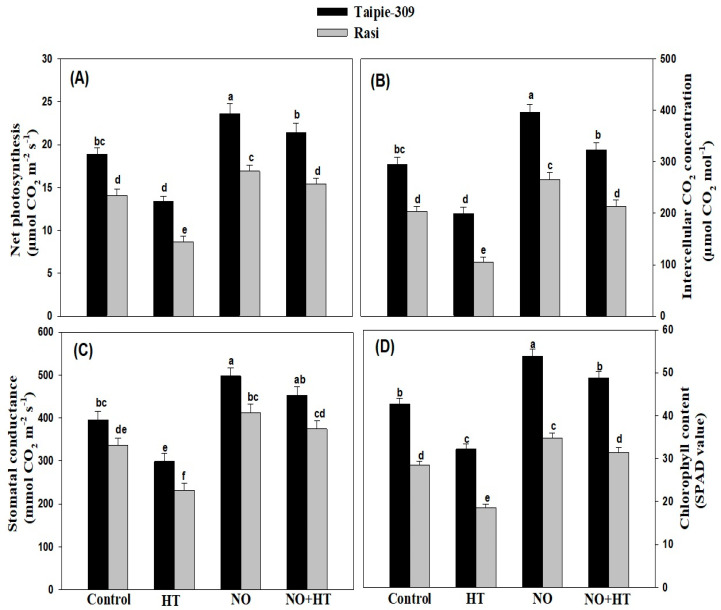
Net photosynthesis (**A**), stomatal conductance (**B**), intercellular CO_2_ concentration (**C**), and chlorophyll content (SPAD value) (**D**) in Taipie-309 and Rasi cultivars of rice (*Oryza sativa* L.) at 30 days after germination (DAG), plants were grown with/without high temperature stress and treated with foliar 100 µM SNP (NO donor). Data are presented as treatments mean ± SE (n = 4). The values followed by same letters above bars represent that data did not differ significantly by LSD test at *p* < 0.05.

High temperature stress significantly decreased net photosynthesis, stomatal conductance, intercellular CO_2_ concentration and chlorophyll content (SPAD value) by 29.1%, 24.4%, 32.3%, and 24.5% in Taipie-309 and 38.2%, 31.0%, 48.3%, and 34.7% in Rasi, respectively compared to the control. Under no stress condition, application of SNP at 100 µM increased the photosynthetic parameters and SPAD in both the cultivars, but to a greater extent in Taipie-309 than Rasi compared to the control. SNP (100 µM) treated plants grown with high temperature proved effective in alleviating high temperature stress in both the cultivars. Taipie-309 was more responsive to SNP and exhibited greater capacity for amelioration and promotion of photosynthetic parameters and SPAD than Rasi. High temperature treated plants in presence of 100 µM SNP showed increase in the above characteristics to 13.2%, 14.4%, 9.46% and 14.2% in Taipie-309 and 9.21%, 11.2%, 5.12%, and 10.1% in Rasi, respectively compared to the control (Figure 3).

**Figure 4:**
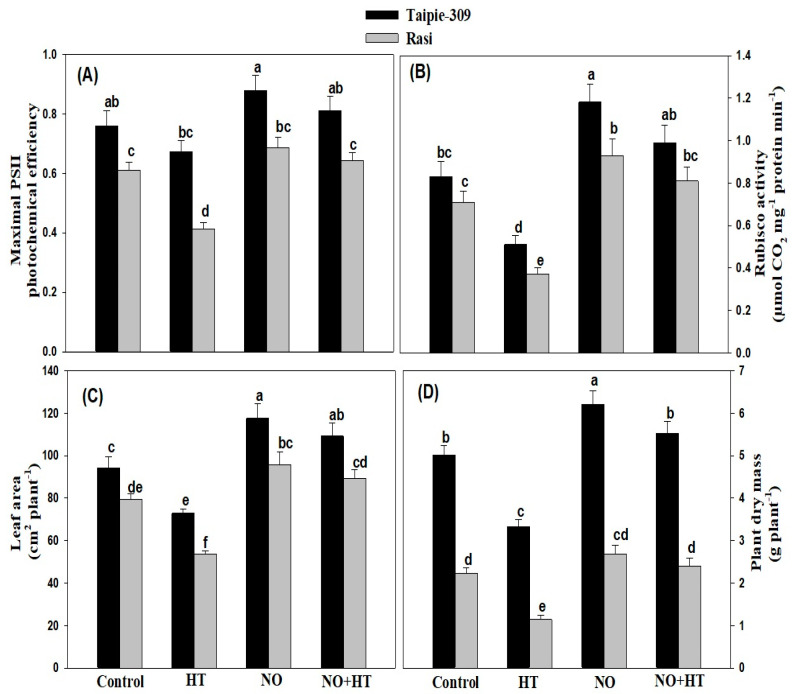
Maximal PSII photochemical efficiency (**A**), Rubisco activity (**B**), leaf area (**C**), and plant dry mass (**D**) in Taipie-309 and Rasi cultivars of rice (*Oryza sativa* L.) at 30 days after germination (DAG), plants were grown with/without high temperature stress and treated with foliar 100 µM SNP (NO donor). Data are presented as treatments mean ± SE (n = 4). The values followed by same letters above bars represent that data did not differ significantly by LSD test at *p* < 0.05.

High temperature stress markedly affected the activity of Rubisco by 11.5% in Taipie-309 and 32.0% in Rasi as compared to the control plants. On the other hand, foliar 100 µM SNP treatment considerably increased Rubisco by 19.2% in Taipie-309 and 14.0% in Rasi under high temperature conditions compared to control plants. High temperature stress significantly reduced PSII activity in both rice cultivars compared to control plants. Application of SNP under high temperature stress inhibited the decrease and improved PSII activity compared to control plants (Figure 4A,B).

Leaf area and plant dry mass were reduced with high temperature treatment in both cultivars, but more prominently in Rasi compared to the control (Figure 4C,D). Under no stress, application of SNP increased leaf area and plant dry mass by 24.7% and 23.7% in Taipie-309 and by 20.8% and 20.1% in Rasi, respectively compared to the control plants. Application of SNP resulted in reduction in adverse effects of high temperature in both cultivars and increased leaf area by 16.0% in Taipie-309 and by 12.7% in Rasi and plant dry mass by 10.1% in Taipie-309 and 7.17% in Rasi compared to the control plants.

### 3.7. NO Increases Nitrogen Content, Nitrate Reductase Activity and Photosynthetic-NUE under High Temperature Stress

**Figure 5:**
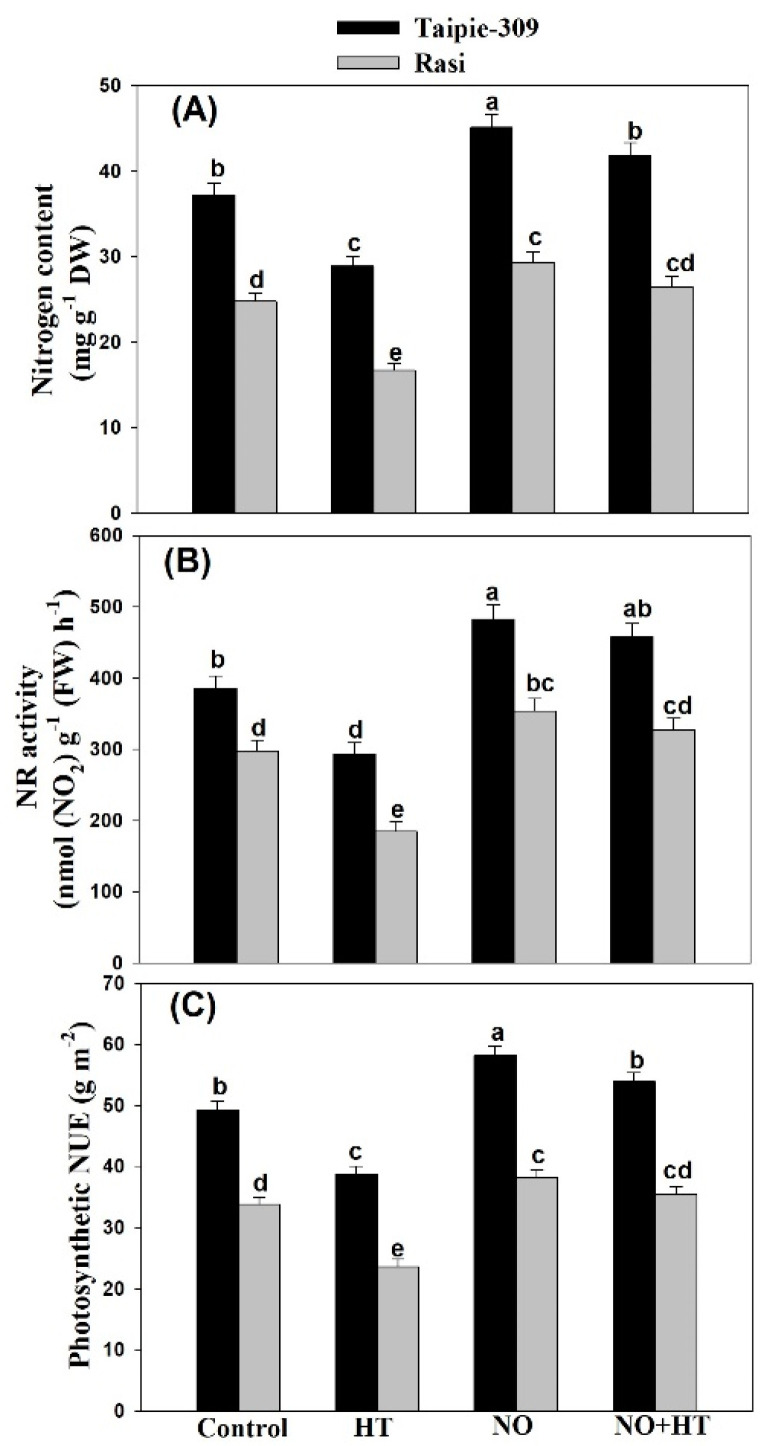
Leaf nitrogen content (**A**), NR activity (**B**), and photosynthetic NUE (**C**) in Taipie-309 and Rasi cultivars of rice (*Oryza sativa* L.) at 30 days after germination (DAG), plants were grown with/without high temperature stress and treated with foliar 100 µM SNP (NO donor). Data are presented as treatments mean ± SE (n = 4). The values followed by same letters above bars represent that data did not differ significantly by LSD test at *p* < 0.05. DW, dry weight; FW, fresh weight; NUE, nitrogen use efficiency.

High temperature stress decreased N content, NR activity and photosynthetic-NUE of both cultivars, but Rasi showed higher decrease than Taipie-309 compared to the control. However, high temperature-induced negative effects were diminished in the SNP-treated plants (Figure 5). Under no stress condition, application of 100 µM SNP increased N content, NR activity and photosynthetic-NUE in both the cultivars, but maximum increase was observed in Taipie-309. An increase in N content by 21.2% and 18.1%, NR activity by 25.1% and 19.0% and photosynthetic-NUE by 18.2% and 13.0% in cultivar Taipie-309 and Rasi, respectively in comparison to the control was registered with the application of 100 µM SNP. High temperature inhibited N content, NR activity and photosynthetic-NUE by 22.3%, 24.0% and 21.3% in Taipie-309 and 32.6%, 37.9%, and 30.1% in Rasi, respectively compared to the control. Supplementation of 100 µM SNP reduced the adverse effects of high temperature and the decrease in N content, NR activity and photosynthetic-NUE was limited to 12.6%, 18.8%, and 9.55% in cultivar Taipie-309 and 6.85%, 9.92%, and 5.02% in cultivar Rasi, respectively compared with the control.

### 3.8. Effect of NO on Sulfur-Assimilation Capacity and Photosynthetic-SUE under High Temperature Stress

**Figure 6:**
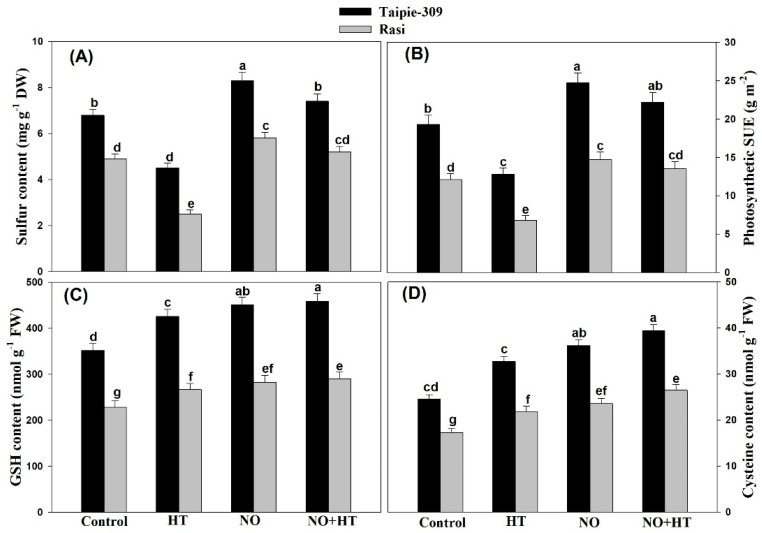
Leaf sulfur content (**A**), photosynthetic SUE (**B**), GSH content (**C**), and Cys content (**D**) in Taipie-309 and Rasi cultivars of rice (*Oryza sativa* L.) at 30 days after germination (DAG), plants were grown with/without high temperature stress and treated with foliar 100 µM SNP (NO donor). Data are presented as treatments mean ± SE (n = 4). The values followed by same letters above bars represent that data did not differ significantly by LSD test at *p* < 0.05. DW, dry weight; FW, fresh weight; SUE, sulfur use efficiency.

The influence of NO was assessed on S-assimilation to monitor its contribution in high temperature stress alleviation. High temperature decreased S content and photosynthetic-SUE by 48.9% and 43.8% to a greater extent in Rasi and 33.8% and 33.6% in Taipie-309, respectively compared to the control. Application of 100 µM SNP increased the S content and photosynthetic-SUE and maximum increase was observed in Taipie-309 than Rasi under no stress and high temperature condition over the control. Application of 100 µM SNP alone increased the S content and photosynthetic-SUE by 22.0% and 27.9% in Taipie-309 and 18.3% and 21.4% in Rasi, respectively compared with the control. Under high temperature condition plant treated with 100 µM SNP reduced the adverse effects of high temperature and increase the S content by 8.82% and 6.12% and photosynthetic-SUE by 15.0% and 11.5% in cultivar Taipie-309 and Rasi, respectively in comparison with the control. High temperature treatment increased both Cys and GSH content in comparison to the control in both the cultivars, but to a greater extent in Taipie-309 than Rasi. NO application at 100 µM SNP resulted in maximal increase in content of Cys by 47.7% and GSH by 28.1% in Taipie-309 while in Rasi, Cys, and GSH content increased only by 36.4% and 23.7%, respectively compared with the control plants. High temperature plus 100 µM SNP treatment increased Cys and GSH content by 60.8% and 30.3% in Taipie-309 and 53.1% and 26.8% in Rasi respectively, compared to the control (Figure 6).

### 3.9. Effect of NO Application on Agronomic Traits under High Temperature Stress

High temperature stress induced significant reduction in number of tillers per plant by (20.5% and 38.5%), number of panicles per plant by (22.5% and 41.6%), panicles length by (8.8% and 17.2%), and number of grain per panicle by (20.8% and 26.9%) in cultivar Taipie-309 and Rasi, respectively, compared to control plants (Table 4).

In addition, SNP-treated plants in no stress and stress condition showed increased number of tillers by (17.5% and 8.5%), number of panicles by (11.8% and 5.6%), panicles length by (6.8% and 2.4%), and number of grains per panicle by (11.0% and 5.3%) in Taipie-309, while in Rasi number of tillers by (12.8% and 5.7%), number of panicles by (8.3% and 3.3%), panicles length by (3.5% and 1.4%), and number of grains per panicle by (12.0% and 3.3%), respectively, compared to control plants.

### 3.10. Effect of NO on Stomatal Response

**Figure 7:**
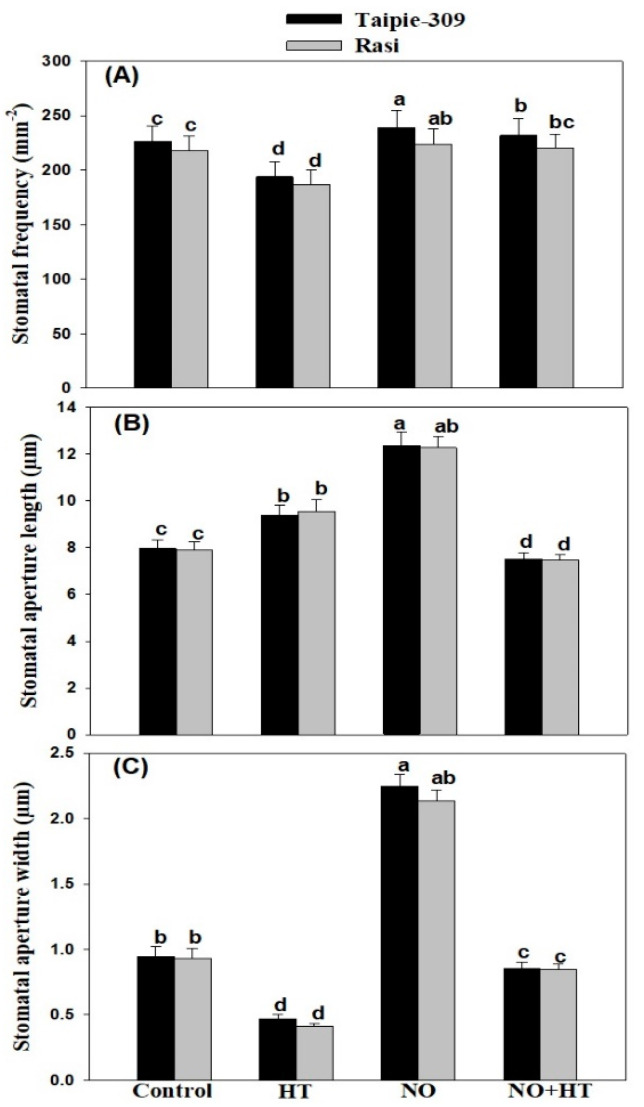
Stomatal frequency (**A**), stomatal aperture length (**B**), and width (**C**) in Taipie-309 and Rasi cultivars of rice (*Oryza sativa* L.) at 30 days after germination (DAG), plants were grown with/without high temperature stress and treated with foliar 100 µM SNP (NO donor). Data are presented as treatments mean ± SE (n = 4). The values followed by same letters above bars represent that data did not differ significantly by LSD test at *p* < 0.05.

To assess the effect of NO on stomatal responses, SEM analysis was done to observe the changes in stomatal behavior of Taipie-309 and Rasi, evaluated on the leaf of both controls and treated plants under high temperature stress. Under control condition, stomatal frequency was higher in Taipie-309 (226 mm^−2^) compared to Rasi (218 mm^−2^), and stomatal apertures (length and width) were 7.96 µm and 0.943 µm in Taipie-309 and 7.92 µm and 0.932 µm in Rasi, respectively. However, the plants treated with high temperature exhibited reduced stomatal frequency in both the cultivars, with 194 mm^−2^ in Taipie-309 and 187 mm^−2^ in Rasi. High temperature-treated plants showed increased stomatal aperture in length by 9.41 µm and 9.55 µm but decreased width of stomatal aperture in both the cultivars Taipie-309 and Rasi, respectively, compared to the control plants. On the other hand, plants supplemented with SNP showed higher stomatal frequency in both the cultivars by 239 mm^−2^ in Taipie-309 and 224 mm^−2^ in Rasi and also exhibited maximum increase in length and width of stomatal aperture by 12.38 µm and 2.246 µm in Taipie-309 and 12.27 µm and 2.137 µm in Rasi, respectively compared to the control plants. In addition to this, under high temperature stress condition, treatment of SNP showed an increase in stomatal frequency by 232 mm^−2^ in Taipie-309 and 220 mm^−2^ in Rasi cultivar, and increase in length and width of stomatal aperture were also recorded in both the cultivars by 7.52 µm and 0.851 µm in Taipie-309 and by 7.48 µm and 0.845 µm in Rasi, respectively compared to the control plants (Figure 7).

### 3.11. Effect of NO Scavenger on Photosynthetic and Growth Parameters

High temperature stress increased H_2_O_2_ content but decreased photosynthetic-NUE and photosynthetic-SUE, photosynthesis and plant dry mass compared to the control plants. Supplementation of NO improved the above characteristics in both the rice cultivars Taipie-309 and Rasi, more conspicuously in Taipie-309 in comparison to the control plants. However, the treatment of cPTIO (NO scavenger) in the presence of SNP and high temperature reversed the effect of SNP and decreased the aforementioned parameters, photosynthetic-NUE by 33.7% and 45.5% and photosynthetic-SUE by 48.7% and 55.0%, net photosynthesis 43.3% and 51.9% and plant dry mass by 49.4% and 60.1% in Taipie-309 and Rasi, respectively compared to the control plants. In the presence of cPTIO, H_2_O_2_ content increased by 128.1% and 148.5% in Taipie-309 and Rasi cultivars as compared to the control plants (Table 5).

## 4. Discussion

The aim of the present study was to evaluate the biochemical and physiological alterations induced by 100 µM SNP (NO donor) in high nutrient-use efficient (Taipie-309) and low nutrient-use efficient (Rasi) rice cultivars under high temperature stress. High temperature stress induces accumulation of ROS, which leads to oxidative damage in plants [40,41]. High temperature stress-mediated structural and functional changes in chloroplast occur as a result of the oxidation of thylakoid membranes and proteins, as was observed by increased MDA levels in maize, rice, and soybean plants [42,43]. In the present study, high temperature stress increased content of H_2_O_2_ and TBARS in both cultivars was lowered by SNP application (Figure 1A,B). However, the extent of lowering the indicators of oxidative stress was comparatively higher in Taipie-309 than Rasi. This protective effect of NO may be associated with its role as antioxidant and by directly scavenging ROS such as O_2_^−^, to form peroxynitrite anion (ONOO^−^), which is less toxic than H_2_O_2_ or by inhibiting NADPH oxide and reducing ROS production [44,45,46]. The earlier studies have shown that NO prevents ROS generation and lipid peroxidation in the plants grown under high temperature stress [47,48,49].

The accumulation of proline is another strategy adopted by plants to combat high temperature stress induced negative impacts of ROS [50,51,52,53] The content of proline was comparatively higher in Taipie-309 than Rasi, which was further enhanced by NO application under heat stress. The studies on broccoli and cucumber under salinity stress [54,55], faba bean and spring maize under high temperature stress [52,56], wheat under osmotic stress [57] and *Cakile maritima* under water deficit stress [58] have shown that NO increased proline production. Proline production was dependent on the NO generation because NO induces the gene pyrroline-5-carboxylate synthase (P5CS) and causes proline accumulation [50,59]. Furthermore, Alamri et al. [52] reported that an equivalent increase in proline content with increasing production of ROS triggered a defense system in plants to scavenge ROS. It was proposed by Alamri et al. [52] that NO not only acted as a signaling molecule but also promoted the biosynthesis of proline in faba bean plant under high temperature stress. Consequently, proline content increased in leaves by NO treatment, thus proline functions by directly scavenging ROS and prevents further lipid peroxidation, thereby increased high temperature stress tolerance.

High temperature stress induces an increased generation of ROS including H_2_O_2_, O_2_^−^ and OH^−^ resulting in oxidative stress and cause damage to cellular constituents, membranes and macromolecules, and disrupt metabolic functions [41,60]. The antioxidant system in plants scavenges excessively accumulated ROS under high temperature stress and restores the impairment induced by ROS [61,62]. The present study showed that the activities of antioxidant enzymes were remarkably higher in Taipie-309 than Rasi under high temperature condition, which were markedly increased by SNP treatment under high temperature stress. Our results are in accordance with the results of previous studies of [48,52,56,63] in *Lablab purpureus*, wheat, faba bean, and spring maize under high temperature stress. Similar responses were observed in mustard, chickpea, tomato, wheat, and pepper under salinity and cadmium stress [18,64,65,66,67]. Akram et al. [54] suggested that NO may activate the stress associated genes and increase the activities of antioxidant enzymes in stressed plants. It is not difficult to conclude that the enhanced antioxidant defense system, as indicated by promoted antioxidant enzymes activities and ROS scavenging ability, account for the high temperature tolerance induced by NO.

The chlorophyll content (SPAD value) was more prominently reduced in Rasi plants exposed to high temperature stress, but the SNP application limited the decrease in chlorophyll content. These results are consistent with the findings of [68] in wheat and [69] in rice plants. Increased chlorophyll content due to NO treatment has already been reported in *Cakile maritima* under water deficit stress [58], in soybean under high temperature stress [16], in mustard under Cd stress [21], in tomato and mustard under salinity stress [19,70], and in mustard under Cu stress [71]. Earlier reports have indicated that exogenously applied SNP improved chlorophyll biosynthesis, as it promoted uptake, translocation and internal availability of iron in plants [72,73,74]. The role of NO in temperature-dependent chlorophyll biosynthesis and Rubisco by overexpressing nitric oxide-associated1 (NOA1) in *O. sativa* plants has also been reported [75]. Similarly, there was decline in other photosynthetic characteristics more conspicuously in Rasi under high temperature stress. The decline in net photosynthesis under high temperature stress may be associated with stomatal closure which reduced the availability of intercellular CO_2_ concentration. Application of SNP resulted in the alleviation of high temperature stress-induced effects on net photosynthesis and maximal PSII photochemical efficiency. In several species such as chryasanthemum [76], soybean [17], rice [69], *Lablab purpureus* [63], and *Festuca arundinacea* [77], high temperature stress-induced decrease in photosynthesis alleviated by SNP treatment has been reported. In the present study, the activity of Rubisco was severely affected by the exposure to high temperature stress. The inhibition of photosynthesis under high temperature stress may be related to the decline of Rubisco activity [78]. Carmo-Silva et al. [79] reported that high temperature stress reduced the availability of CO_2_ resulting in the decreased activity of Rubisco that limited the photosynthesis. However, the application of SNP significantly enhanced the activity of Rubisco, but the maximum enhancement was observed in Taipie-309 (Figure 4b). According to Song et al. [78] the effect of NO on photosynthesis in heat stress condition might be mediated by regulating Rubisco activity through S-nitrosylation of Cys-residue.

Stomata are important for plant survival under stress, and the drastic temperature increase may cause stomatal closure in different plant species to reduce transpirational water loss in order to increase thermotolerance [80,81]. However, Kostaki et al. [82] have shown that high temperature promotes guard cell expansion which opens stomatal pores to facilitate leaf cooling. High temperature stress resulted in a variation in the stomatal aperture and stomatal frequency compared with the control plants. Our results showed that high temperature stress induced stomatal closure and increased stomatal aperture but width was reduced in both cultivars. However, the exogenous application of SNP under high temperature stress reduced the decrease in stomatal aperture and increased stomatal frequency. Several studies provide evidence to support SNP mediated stomatal opening and increase in stomatal aperture [19,20,71]. Jahan et al. [20] showed NO application increased stomatal frequency under with and without stress condition. Similarly, the stomatal apertures were found clearly open in SNP supplied plants compared to the control or Cd-treated mustard plants [22].

In the present study, application of SNP increased S-assimilation more efficiently in Taipie-309 than Rasi under high temperature stress. The high temperature tolerance of Taipie-309 may be attributed to its ability to utilize maximum S available and representing highest photosynthetic-SUE under high temperature stress. Moreover, the higher S-assimilation resulted in the formation of more GSH, as it is required for reducing oxidative stress and alleviation of high temperature-induced damages in plants. Furthermore, the content of GSH has been reported to increase by NO in mustard in presence and absence of Cu stress [71]. Similarly, GSH content was significantly increased by SNP supplementation in wheat under high temperature stress [48]. Thus, exogenous NO treatment could participate in the synthesis or regeneration of GSH under high temperature stress. Nitrate reductase is a key enzyme that catalyzes the first reaction in nitrate assimilation, the reduction of nitrate to nitrite, which is important for nitrogen acquisition by crop plants and for handling its signaling role in different plant processes [83]. Onwueme et al. [84] concluded that NR seems to be unique among the enzymes studied in being inactivated by heat stress. In our study, NR activity decreased under high temperature stress in both cultivars. This is consistent with the previous studies of Khan et al. [68] in wheat under high temperature stress, Manai et al. [65] in tomato under salinity stress, and Hasan et al. [85] in mungbean under Cd stress. In addition, NO can regulate NR activity [86] as has been found in the present study and also by Manai et al. [65] who found that the exogenous NO application through the root system stimulated the NR activity in both roots and leaves which alleviated the negative effects of salinity stress. Majeed et al. [87] reported significant increase in NR activity of 100 µM SNP treated maize plants under drought stress. In the present study, high temperature stress significantly decreased the leaf N content and photosynthetic-NUE of Taipie-309 and Rasi, but exogenous SNP application elevated leaf N content and photosynthetic-NUE in both cultivars under heat stress. This is consistent with the previous studies of Jahan et al. [20] in mustard under salinity stress and Hasan et al. [85] in mung bean under Cd stress.

High temperature treatment has been shown to enhance the endogenous levels of NO. These results are in agreement with the findings of Gould et al. [88] and Kaur and Kaur [56]. Kaur and Kaur [56] suggested that the higher NO content under high temperature stress acts as an adaptive mechanism in tolerating extreme temperatures in maize cultivar. Rather et al. [71] found that exogenous SNP application resulted in the reduction of NO generation in plants grown with or without Cu stress.

Tiller production in rice is an important agronomical trait and is very sensitive to temperature [89]. It has been reported that temperatures above 33 °C were unfavorable for tillering and the number of tillers reduced in high temperature conditions [90,91]. In addition, high temperature stress decreased leaf area, panicle length, and the relative water content of spikes [92] and reduced plant height, dry weight, and tiller number [93]. Fahad et al. [94] reported that under high temperature conditions rice plant developed less leaf area and substantial reduction in leaf area resulted in less photosynthesis with concomitant decline in grain yield [95]. In the present study, the application of SNP reduced the negative effects of high temperature stress on leaf area, plant dry mass, number of tillers and panicles per plant, length of panicle, and number of grains per panicle. However, more pronounced effect of SNP was observed in cultivar Taipie-309. It has been reported that exogenous application of plant growth regulators increased thermo-tolerance and provided better rice performance under high temperature condition [96].

The Taipie-309, a high photosynthetic-N and SUE cultivar exhibited higher inherent tolerance mechanisms and was more responsive to NO application than the other low photosynthetic-N and SUE cultivar Rasi. The ability of Taipie-309 to have higher N and S assimilation for greater incorporation of N and S into photosynthetic enzymes such as Rubisco and GSH helped the plants to maintain higher photosynthesis under high temperature stress. Moreover, NO induced the assimilation of N and S was more prominently in Taipie-309. The NO induced activity of ascorbate-glutathione cycle enzymes and produced higher content of GSH more prominently in Taipie-309 which led to higher tolerance of this cultivar to high temperature stress. As the Taipie-309 had higher N and S, it also contributed to higher amount of GSH. These mechanisms collectively conferred higher tolerance to Taipie-309, which was primarily based on the use efficiency of nutrients. The study found support from the use of cPTIO, NO scavenger. Supplementation of cPTIO provides the evidence that NO is central to the improvement of photosynthesis inhibited by high-temperature induced stress. The cPTIO treated plants had higher content of H_2_O_2_ in presence of high temperature stress with reduction in photosynthetic N and SUE and plant dry mass of plants. This substantiated the role of SNP in promoting photosynthesis and growth of plants inhibited by high temperature stress (Table 5). It was previously found that cPTIO-treated plants in the presence of SNP under abiotic stresses such as Cd and salt resulted in a decrease in plant dry mass and leaf area and an increase in oxidative stress parameters [22,97].

## 5. Conclusions

In conclusion, the present findings suggest that the high photosynthetic-NUE and SUE cultivar responded more positively to SNP than the low photosynthetic-NUE and SUE rice cultivar. Application of SNP significantly reversed high temperature stress-induced photosynthetic inhibition in rice cultivars which were related to changes in Rubisco activity, PSII activity, and photosynthetic-NUE and SUE. The exogenous SNP increased high temperature tolerance and prominently mitigated adverse effects imparted by high temperature stress in rice cultivars through modulating antioxidant defense system. Thus, NO might function as an antioxidant by directly scavenging reactive oxygen species and activating ascorbate-glutathione cycle and GSH synthesis. The effect of SNP application in inducing physiological processes was more conspicuous in cultivar Taipie-309 than Rasi. The use of cPTIO (NO scavenger) confirmed that NO derived from SNP was responsible for alleviation of high temperature stress.

## Figures and Tables

**Table 1:** Leaf nitrogen and sulfur content, photosynthetic nitrogen-use efficiency (NUE), photosynthetic sulfur-use efficiency (SUE), plant dry mass, and leaf area in cultivars of rice (*Oryza sativa* L.) at 30 days after germination (DAG).

Cultivars	Nitrogen Content (mg g^−1^ DW)	Sulfur Content (mg g^−1^ DW)	Photosynthetic- NUE (g m^−2^)	Photosynthetic- SUE (g m^−2^)	Plant Dry Mass (g Plant^−1^)	Leaf Area (cm^2^ plant^−1^)
BPT-5204	34.5 ± 1.27 ^ab^	6.5 ± 0.30 ^ab^	46.3 ± 1.44 ^ab^	17.1 ± 1.23 ^abc^	4.44 ± 0.18 ^b^	91.5 ± 4.95 ^ab^
Taipie-309	37.2 ± 1.41 ^a^	6.8 ± 0.35 ^a^	49.2 ± 1.50 ^a^	19.3 ± 1.32 ^a^	5.02 ± 0.20 ^a^	94.2 ± 5.35 ^a^
Pusa-44	28.6 ± 1.15 ^cd^	5.8 ± 0.20 ^bcde^	38.6 ± 1.34 ^d^	14.7 ± 1.09 ^bcde^	3.91 ± 0.17 ^cd^	83.6 ± 3.70 ^ab^
Panvel	27.4 ± 1.11 ^cd^	5.6 ± 0.17 ^cdef^	38.1 ± 1.30 ^de^	14.4 ± 1.06 ^bcde^	4.21 ± 0.18 ^bc^	82.7 ± 3.65 ^ab^
Rasi	24.8 ± 0.95 ^d^	4.9 ± 0.1 ^f^	33.8 ± 1.21 ^e^	12.1 ± 0.85 ^e^	2.23 ± 0.13 ^g^	79.3 ± 2.70 ^b^
MTU-1010	29.3 ± 1.20 ^c^	6.0 ± 0.26 ^bcd^	40.9 ± 1.31 ^cd^	15.2 ± 1.17 ^bcde^	3.67 ± 0.16 ^de^	86.8 ± 4.27 ^ab^
CR-DHAN-310	35.7 ± 1.33 ^ab^	6.5 ± 0.30 ^ab^	47.5 ± 1.47 ^ab^	17.8 ± 1.28 ^ab^	4.68 ± 0.19 ^ab^	89.7 ± 4.79 ^ab^
Nidhi	26.8 ± 1.08 ^cd^	5.3 ± 0.15 ^def^	37.4 ± 1.27 ^de^	13.9 ± 0.98 ^cde^	3.11 ± 0.14 ^f^	81.8 ± 3.27 ^ab^
CR-Dhan-311	26.1 ± 1.05 ^cd^	5.1 ± 0.11 ^ef^	36.8 ± 1.24 ^de^	13.2 ± 0.95 ^de^	3.03 ± 0.13 ^f^	81.2 ± 3.21 ^ab^
Nagina-22	32.9 ± 1.25 ^d^	6.2 ± 0.28 ^abc^	43.7 ± 1.37 ^bc^	16.7 ± 1.21 ^abcd^	3.26 ± 0.15 ^ef^	87.4 ± 4.45 ^ab^

Data are presented as treatments mean ± SE (n = 4). Data followed by same letter are not significantly different by LSD test at *p* < 0.05. DAG: Days after germination.

**Table 2:** Net photosynthesis, intercellular CO_2_ concentration, stomatal conductance, chlorophyll content (SPAD value) and maximal PSII photochemical efficiency in cultivars of rice (*Oryza sativa* L.) at 30 days after germination (DAG). Data are presented as treatments mean ± SE (n = 4). Data followed by same letter are not significantly different by LSD test at *p* < 0.05.

Cultivars	Net Photosynthesis (µmol CO_2_ m^−2^ s^−1^)	Intercellular CO_2_ Concentration (µmol CO_2_ mol^−1^)	Stomatal Conductance (mmol CO_2_ m^−2^ s^−1^)	Chlorophyll Content (SPAD Value)	Maximal PSII Photochemical Efficiency
BPT-5204	17.7 ± 0.81 ^abc^	275.2 ± 13.06 ^ab^	383.8 ± 19.13 ^abc^	40.9 ± 1.25 ^ab^	0.711 ± 0.041 ^ab^
Taipie-309	18.9 ± 0.88 ^a^	294.8 ± 13.72 ^a^	395.5 ± 19.28 ^a^	42.7 ± 1.36 ^a^	0.76 ± 0.049 ^a^
Pusa-44	16.1 ± 0.65 ^bcdef^	249.4 ± 11.97 ^bcd^	366.7 ± 18.34 ^abcde^	34.7 ± 1.20 ^cd^	0.688 ± 0.035 ^ab^
Panvel	15.7 ± 0.51 ^cdef^	232.6 ± 11.50 ^cde^	360.4 ± 18.25 ^bcde^	33.2 ± 1.13 ^de^	0.678 ± 0.034 ^ab^
Rasi	14.1 ± 0.37 ^f^	203.1 ± 10.25 ^e^	336.5 ± 17.29 ^e^	28.5 ± 0.9 ^f^	0.609 ± 0.029 ^b^
MTU-1010	16.8 ± 0.66 ^abcde^	255.7 ± 12.48 ^abcd^	371.8 ± 18.53 ^abcde^	35.6 ± 1.16 ^cd^	0.701 ± 0.036 ^ab^
CR-Dhan-310	18.0 ± 0.85 ^ab^	280.3 ± 13.32 ^ab^	389.2 ± 19.16 ^ab^	38.2 ± 1.21 ^bc^	0.736 ± 0.046 ^ab^
Nidhi	15.2 ± 0.47 ^def^	230.2 ± 10.95 ^cde^	353.1 ± 18.15 ^cde^	31.9 ± 1.10 ^def^	0.632 ± 0.031 ^ab^
CR-Dhan-311	14.9 ± 0.41 ^ef^	217.4 ± 10.47 ^de^	348.6 ± 18.12 ^de^	30.4 ± 1.05 ^ef^	0.644 ± 0.031 ^ab^
Nagina-22	17.3 ± 0.70 ^abcd^	266.0 ± 12.91 ^abc^	377.3 ± 18.90 ^abcd^	37.9 ± 1.20 ^bc^	0.705 ± 0.037 ^ab^

**Table 3:** Number of tillers per plant, number of panicles per plant, panicles length, and number of grains per panicle in cultivars of rice (*Oryza sativa* L.) at harvest.

Cultivars	Number of Tillers/Plant	Number of Panicles/Plant	Panicles Length (cm)	Number of Grains/Panicle
BPT-5204	15 ± 0.60 ^c^	15 ± 0.63 ^ab^	22.0 ± 0.87 ^b^	153 ± 11.0 ^abc^
Taipie-309	20 ± 0.66 ^a^	16 ± 0.65 ^a^	25.0 ± 0.90 ^a^	180 ± 12.0 ^a^
Pusa-44	10 ± 0.58 ^d^	10 ± 0.56 ^de^	19.3 ± 0.78 ^c^	157 ± 11.2 ^ab^
Panvel	8 ± 0.47 ^ef^	11 ± 0.58 ^cd^	19.1 ± 0.72 ^c^	142 ± 10.4 ^bc^
Rasi	7 ± 0.41 ^f^	6 ± 0.49 ^f^	13.9 ± 0.65 ^f^	98 ± 9.33 ^d^
MTU-1010	9 ± 0.51 ^de^	14 ± 0.61 ^b^	20.3 ± 0.83 ^bc^	148 ± 10.6 ^abc^
CR-Dhan-310	18 ± 0.65 ^b^	15 ± 0.62 ^ab^	22.2 ± 0.89 ^b^	165 ± 11.6 ^ab^
Nidhi	8 ± 0.46 ^ef^	9 ± 0.55 ^e^	15.7 ± 0.66 ^ef^	103 ± 9.41 ^d^
CR-Dhan-311	9 ± 0.52 ^de^	9 ± 0.55 ^e^	16.5 ± 0.68 ^de^	121 ± 9.93 ^cd^
Nagina-22	9 ± 0.52 ^de^	12 ± 0.60 ^c^	18.3 ± 0.70 ^cd^	130 ± 10.1 ^bcd^

Data are presented as treatments mean ± SE (n = 4). Data followed by same letter are not significantly different by LSD test at *p* < 0.05.

**Table 4:** Number of tillers per plant, number of panicles per plant, panicles length, and number of grains per panicle in Taipie-309 and Rasi cultivars of rice (*Oryza sativa* L.) at harvest time, plants were grown with/without high temperature (HT) stress and treated with foliar 100 µM SNP (NO donor).

Cultivar	Treatments	Number of Tillers per Plant	Number of Panicles per Plant	Panicles Length (cm)	Number of Grains per Panicle
Taipie-309	Control	20.0 ± 0.66 ^c^	16.0 ± 0.43 ^b^	25.0 ± 0.90 ^ab^	180.0 ± 12.0 ^a^
	HT	15.9 ± 0.60 ^d^	12.4 ± 0.40 ^c^	22.8 ± 0.86 ^b^	142.5 ± 11.3 ^b^
	NO	23.5 ± 0.69 ^a^	17.9 ± 0.60 ^a^	26.7 ± 0.97 ^a^	199.9 ± 15.5 ^a^
	NO + HT	21.7 ± 0.65 ^b^	16.9 ± 0.50 ^ab^	25.6 ± 0.92 ^a^	189.7 ± 13.5 ^ab^
Rasi	Control	7.0 ± 0.41 ^e^	6.0 ± 0.32 ^d^	13.9 ± 0.66 ^cd^	98.0 ± 9.33 ^cd^
	HT	4.3 ± 0.30 ^f^	3.5 ± 0.23 ^e^	11.5 ± 0.55 ^d^	71.6 ± 7.40 ^d^
	NO	7.9 ± 0.51 ^e^	6.5 ± 0.40 ^d^	14.4 ± 0.72 ^c^	109.8 ± 10.9 ^bc^
	NO + HT	7.4 ± 0.45 ^e^	6.2 ± 0.35 ^d^	14.1 ± 0.70 ^c^	101.3 ± 10.2 ^cd^

Data are presented as treatments mean ± SE (n = 4). The values followed by same letters above bars represent that data did not differ significantly by LSD test at *p* < 0.05.

**Table 5:** H_2_O_2_ content (nmol g^−1^ FW), plant dry mass (g plant^−1^), net photosynthesis (µ mol CO_2_ m^−2^ s^−1^), photosynthetic NUE (g m^−2^) and photosynthetic-SUE (g m^−2^) in Taipie-309 and Rasi cultivars of rice (*Oryza sativa* L.) plants grown with/without high temperature (HT) stress and treated with foliar 100 µM SNP (NO donor) and 100µM cPTIO at 30 days after germination (DAG).

Cultivar	Treatments	H_2_O_2_ Content	Photosynthetic-NUE	Photosynthetic-SUE	Net Photosynthesis	Plant Dry Mass
Taipie-309	Control	47.6 ± 2.6 ^h^	49.2 ± 1.50 ^ab^	19.3 ± 1.21 ^b^	18.9 ± 0.76 ^b^	5.02 ± 0.22 ^ab^
	HT	104.9 ± 4.74 ^cd^	38.7 ± 1.34 ^c^	12.8 ± 0.95 ^cd^	13.4 ± 0.71 ^cde^	3.32 ± 0.185 ^c^
	NO + HT	56.7 ± 3.66 ^fg^	52.3 ± 1.50 ^a^	22.2 ± 1.23 ^a^	20.7 ± 1.27 ^a^	5.53 ± 0.27 ^a^
	NO + HT + cPTIO	108.6 ± 4.89 ^c^	32.6 ± 1.12 ^ef^	9.9 ± 0.78 ^f^	10.72 ± 0.67 ^f^	2.54 ± 0.12 ^d^
Rasi	Control	59.8 ± 2.9 ^f^	33.8 ± 1.21 ^e^	12.1 ± 0.93 ^cde^	14.1 ± 0.68 ^cd^	2.23 ± 0.127 ^ef^
	HT	141.2 ± 5.15 ^ab^	23.6 ± 1.93 ^g^	6.8 ± 0.64 ^g^	8.7 ± 0.60 ^g^	1.14 ± 0.97 ^g^
	NO + HT	73.7 ± 4.18 ^e^	35.2 ± 1.85 ^cd^	13.5 ± 0.94 ^c^	15.1 ± 0.71 ^c^	2.39 ± 0.19 ^de^
	NO + HT + cPTIO	148.6 ± 5.26 ^a^	18.4 ± 1.67 ^h^	5.44 ± 0.53 ^h^	6.78 ± 0.56 ^h^	0.89 ± 0.45 ^h^

The superscript letters denote the level of values, starting with a as the highest value and f as the lowest. Data are presented as treatment mean ± SE (n = 4). Data followed by same letter are not significantly different by LSD test at *p* < 0.05.

## Data Availability

The data presented in this study are available in the graphs and tables provided in the manuscript.

## Supplementary Materials

The following are available online at https://www.mdpi.com/2218-273X/11/2/305/s1, File S1: Details of methodology and Table S1 and Table S2: ANOVA Model for tables and figures.
