# Supplementary material for: Nitric Oxide Enhances Photosynthetic Nitrogen and Sulfur-Use Efficiency and Activity of Ascorbate-Glutathione Cycle to Reduce High Temperature Stress-Induced Oxidative Stress in Rice (Oryza sativa L.) Plants
Source: Biomolecules. 2021 Feb 18;11(2):305. doi: 10.3390/biom11020305 (PMC7922496; doi:10.3390/biom11020305)
Supplement: Supplementary file 1 [file biomolecules-11-00305-s001.pdf]

File S1

Details of Methodology

Assay of Rubisco activity

Fresh leaf tissue (1.0 g) was homogenized in ice-cold extraction buffer (0.25 M Tris-HCl; pH 7.8, 0.05 M $MgCl_2$, 0.0025 M EDTA and 37.5 mg DTT) using pre-chilled mortar and pestle. Homogenate was centrifuged at 10,000 × g for 10 min at 4°C, the supernatant was subjected with 100 mM Tris-HCl (pH 8.0) and reaction mixture included 40 mM $NaHCO_3$, 10 mM $MgCl_2$, 0.2 mM NADH, 4 mM ATP, 0.2 mM EDTA, 5 mM DTT, 1 U of glyceraldehyde-3-phosphodehydrogenase, 1 U of 3-phosphoglycerate kinase and 0.2 mM ribulose 1, 5-bisphosphate.

Estimation of proline

Fresh leaf tissue (300 mg) was homogenized in 3 ml of 3% sulphosalicylic acid using mortar and pestle. 1 ml each of acid ninhydrin and glacial acetic acid was added to the homogenate filtrate. Then, reaction was carried for 1 h in a test tube placed in a water bath at 100°C, then the solution was cooled and the reaction was terminated. The mixture was extracted using 4 ml toluene mixed vigorously for few seconds and the OD value was measured in a spectrophotometer (UV-vis L164, Elico, New Delhi) for colored components at 520 nm using L-proline as a standard.

Determination of TBARS

Fresh leaf tissue (500 mg) was homogenized in 0.25% 2-thiobarbituric acid in 10% trichloroacetic acid using mortar and pestle. Homogenized mixture was heated at 95°C for 30 min and then the mixture was cooled on ice bath and centrifuged at 10,000 × g for 10 min. The absorbance of the supernatant was read at 532 nm and corrected for non-specific turbidity by subtracting the absorbance of the same at 600 nm. The amount of thiobarbituric acid reactive substances (TBARS) was calculated using an extinction coefficient (155 mM^{-1} cm^{-1}).

Determination of H_2O_2

Fresh leaf tissue (500 mg) was homogenized in pre-cooled 200 mM perchloric acid ($HClO_4$) using mortar and pestle, followed by centrifugation at 1200 × g for 10 min. Centrifuged supernatant containing perchloric acid was neutralized with 4 M KOH, followed by further centrifugation at 500 × g for 3 min to remove insoluble potassium perchlorate ($KClO_4$). In a final volume of 1.5 ml, the content of reaction mixture were 1 ml of eluate, 400 µl of 12.5 mM 3-(dimethylamino) benzoic acid in 0.375 M phosphate buffer (pH 6.5), 80 µl of 3-methyl-2benzothiazoline hydrazone and 20 µl of peroxidase (0.25 unit). Further peroxidase was added at 25°C to start reaction mixture and the increase in absorbance was recorded at 590 nm for 3 min. Later, H_2O_2 content was calculated using an extinction coefficient (0.28 μmol^{-1} cm^{-1}).

Assay of antioxidant enzymes activities

Activity of APX (EC 1.11.1.11) was determined according to the method described by Nakano and Asada (1981) by recording the decrease in absorbance of ascorbate at 290 nm. The assay mixture contained phosphate buffer (50 mM, pH 7.0), 0.1 mM EDTA, 0.5 mM ascorbate, 0.1 mM H_2O_2 and the enzyme extract. The activity of APX was calculated by using the extinction coefficient 2.8 mM^{-1} cm^{-1}. One unit of enzyme was the amount necessary to decompose 1 µmol of substrate per min at 25°C.

Activity of SOD (EC 1.15.1.1) was determined according to the methods of Beyer and Fridovich (1987) and Giannopolitis and Ries (1977) measured on the basis of potential of the enzyme to prevent the photochemical reduction of nitro blue tetrazolium chloride (NBT). A (5.0 ml) of reaction mixture containing 5.0 mM HEPES (pH 7.6), 50 mM Na_2CO_3 (pH 10.0), 0.1 mM EDTA, 0.025% (v/v) Triton X-100, 13 mM methionine, 1.3 μmol riboflavin and 63 μmol NBT. The enzyme extract was illuminated for 15 min (360 μmol m^{-2} s^{-1}) and a control set was not illuminated to correct the turbidity of background absorbance. A unit of SOD was defined as the amount of enzyme required to cause 50% inhibition of the reaction of NBT at 560 nm.

Activity of GR (EC 1.6.4.2) was determined by the method of Foyer and Halliwell (1976) by monitoring the glutathione dependent oxidation of nicotinamide adenine dinucleotide phosphate (NADPH) at 340 nm. The assay mixture contained phosphate buffer (25 mM, pH 7.8), 0.5 mM oxidized glutathione (GSSG), 0.2 mM NADPH, and the enzyme extract. The activity of GR was calculated by using extinction coefficient 6.2 mM^{-1} cm^{-1}. One unit of enzyme is the amount necessary to decompose 1 μmol of NADPH per min at 25°C.

Nitrate reductase activity

Fresh leaf tissue (1.0 g) was grinded in liquid N_2 using chilled mortar and pestle and then powder was stored at -80°C. The powder was thawed for 10 min. at 4°C and homogenized in Tris-HCl buffer (250 mM and pH 8.5) containing 1.0 mM DTT, 1.0 mM EDTA, 20 mM FAD, 10 mM cysteine and 10% (v/v) glycerol. Homogenate was centrifuged at 10,000 × g for 30 min. at 4°C. Using the procedure of (Nakagawa et al., 1984) NR activity was measured as the rate of nitrite production at 28 C. The assay mixture contained 10 mM KNO_3, HEPES (0.065 M, pH 7.0), 0.5 mM NADH in phosphate buffer (0.04 mM, pH 7.2) and the enzyme extract in the final volume of 1.5 ml. NADH was added for initiating the reaction, incubated for 15 min. and the reaction was terminated by adding 1.0 ml of 1.0 N HCl solution containing 1% sulphanilamide followed by the addition of 1.0 ml of 0.02% aqueous N-1-napthylethylene-di-amine-dihydrochloride. The absorbance was read at 540 nm after 10 min.

Table S1. ANOVA Model for Tables 1–3.

**Source of variation**	**df**	**Sum of squares**	**Mean square**	**F-value**	**Significance** ***p < 0.05***
Replications	3				
Cultivars	9				
Error	27				
Total	39				

Table S2. ANOVA Model for Tables 4-5 and Figures 1–7.

**Source of variation**	**df**	**Sum of squares**	**Sum of square**	**F-value**	**Significance** ***p < 0.05***
Replications	3				
Treatment	7				
Error	21				
Total	31				

Table S3. Calculated values of Fv in Taipie-309 and Rasi cultivars of rice (*Oryza sativa* L.) at 30 DAG, plants were grown with/without high temperature stress and treated with foliar 100 μM SNP (NO donor). Data are presented as treatments mean ± SE (n = 4).

**Cultivars**	**Treatments**	**Maximal fluorescence (Fm)**	**Minimal fluorescence (Fo)**	**Fv = Fm – Fo**
**Taipie-309**	Control	3242	778	2464
	HT	3126	1024	2102
	NO	5242	634	4608
	NO+HT	4179	789	3390
**Rasi**	Control	2340	915	1425
	HT	1526	895	631
	NO	2557	799	1758
	NO+HT	1851	659	1192
